# The protective effects of grape seed oil on induced osteoarthritis of the knee in male rat models

**DOI:** 10.1186/s13018-020-01932-y

**Published:** 2020-09-10

**Authors:** Nader Tanideh, Soheil Ashkani-Esfahani, Farid Sadeghi, Omid Koohi-Hosseinabadi, Cambyz Irajie, Aida Iraji, Bart Lubberts, Soleiman Mohammadi Samani

**Affiliations:** 1 Stem cells Technology Research Center, Shiraz University of Medical Sciences, Shiraz, Iran; 2 Department of Pharmacology, School of Medicine, Shiraz University of Medical Sciences, Shiraz, Iran; 3 Department of Orthopaedic Surgery, Massachusetts General Hospital, Harvard Medical School, Boston, MA USA; 4 Foot and Ankle Research and Innovation Laboratory, Massachusetts General Hospital, Harvard Medical School, Boston, MA USA; 5 Student Research Committee, Shiraz University of Medical Sciences, Shiraz, Iran; 6 Central Research Laboratory, Shiraz University of Medical Sciences, Shiraz, Iran; 7 Department of Medical Biotechnology, School of Advanced Medical Sciences and Technologies, Shiraz University of Medical Sciences, Shiraz, Iran; 8 Center of Nanotechnology in Drug Delivery, Faculty of Pharmacy, Shiraz University of Medical Sciences, Shiraz, Iran

**Keywords:** Osteoarthritis, Grape seed oil, Anti-inflammation, Anti-oxidant

## Abstract

**Background:**

Osteoarthritis (OA), though being treated via various methods and medicines, is still a major healthcare concern mostly due to the increase in diagnosis of these age-related diseases. The present study aimed at investigating the effects of oral and intra articular injection of grape seed oil on OA in male rat models.

**Methods and materials:**

Seventy male rats were selected and their anterior cruciate ligament was cut to induce OA. They were divided into 7 groups (*n* = 10): C1, no treatment; C2, receiving 300 mg/day of Piascledine per os (PO); C3, 1 mg sodium hyaluronate intra-articularly in days 1, 7, 14; C4, 1 mg methyl-prednisolone acetate intra-articularly; E1, avocado and grape seed oil combination (2:1, 300 mg/day) PO; E2, 500 mg/day of grape seed oil PO; E3, 200 mg/day grape seed oil intra-articularly. After 10 weeks, the rats were anesthetized and evaluated radiologically and histopathologically. *P* value ≤ 0.05 was considered as statistically significant.

**Results:**

All the groups made significant differences with C1 regarding all inspected radiological criteria (*P* ≤ 0.05). E1 and E3 showed significantly better effects on medial femoral condyle, medial tibial condyle, joint space width, total osteophyte, and OA scores (*P* ≤ 0.04).

Joint surface, matrix, cell distribution, cell population viability, calcification, and subchondral bone in treatment groups had significantly better scores versus C1 (*P* ≤ 0.04). E1 and E3 had significantly superior results regarding joint surface, cell viability, and calcification (*P* ≤ 0.04).

**Conclusions:**

Grape seed oil has protective effects, both in injectable form and PO in combination with avocado, on OA in rats. Further clinical trials are necessary.

## Introduction

Knee osteoarthritis (OA) is a degenerative joint disease and a dominant cause of pain and disability; OA is described as a slow development disease that is mostly diagnosable in its terminal phase [1]. About 10% of men and 13% of women at the age of over 60 years present with symptoms of knee OA. These symptoms mostly include morning stiffness, joint pain, and joint deformities that affect the quality of life and functional outcomes [2, 3]. To date, there is no definite treatment for this chronic disease and most of the therapeutic methods are focused on symptom management and in severe cases, joint replacement is considered [4, 5]. Previous experiments concluded that the prevention of anti-inflammatory and pro-inflammatory pathways as well as reducing oxidative stress may lead to controlling the development of the OA [6, 7]. Numerous studies found that some herbal medicines due to their immune modulating, anti-inflammatory, and antioxidative effects can prevent OA in experimental models. These medicines are increasingly becoming popular for the treatment of OA symptoms, particularly in experiments and research [8, 9]. In populations with traditional beliefs—i.e., some areas in the middle east countries such as Iran, the will for using traditional and natural medicines gave us the idea to work on some of these medicines that are believed to be effective among them in order to introduce effective and cost-benefit treatments for those suffering from OA and its symptoms.

Grape seed is well known for its variety of anti-oxidative derivatives such as oligomeric proanthocyanidins and catechins, as well as anti-inflammatory potentials, is one of the most popular remedies for the treatment of OA [10, 11]. Literature reported that the efficacy of grape seed extract administered orally on OA in rats led to the prevention of loss of chondrocyte cells and proteoglycan, formation of osteophytes, reducing reactive oxygen species, and inflammation mediators such as MMP13, nitrotyrosine, and IL-1β, in experimental models [8, 12]. The purpose of the present study was to determine the effects of grape seed oil treatment for the prevention of knee OA by evaluating radiological and histopathological outcomes.

## Materials and methods

### Grape seed oil material and extraction

In this study, different attempts were made to prepare emulsions of grape seed oil (Pietro Coricelli™, Italy) with various combinations of the constituents. Out of these attempts, the following three series of emulsions were prepared with different hydrophilic-lipophilic balance (HLB) (HLB: 9, 10, 11, 12). Emulsification was performed using the following procedures. Initially, grape seed oil was transferred to the beaker and heated at 20 °C, then water, as the solvent, was added. Subsequently, the emulsifier was added under intense agitation at 500 rpm for 5 min. Then, in the case of groups I and II, as described below, the mixture was mixed for an extra 25 min; however, group III was completely digested by applying 10 min ultrasonic homogenizer. Finally, the mixture was sterilized by autoclave (121 °C for 15 min) and the emulsion was packaged under a laminar hood with aseptic conditions.

I) Tween 80 (as a nonionic emulsifier in the aqueous phase)/Span 20 (as an emulsifier of lipid-containing compounds)/stirring:

A: 25 g grape seed oil + 23 g water 15% + 0.9375 Span 20 + 0.0625 of Tween 80

B: 25 g grape seed oil + 23 g water 15% + 0.78 Span 20 + 0.22 of Tween 80

C: 25 g grape seed oil + 23 g water 15% + 0.625 Span 20 + 0.375 of Tween 80

D: 25 g grape seed oil + 23 g water 15% + 0.42 Span 20 + 0.53 of Tween 80

II) Tween 80/Span 60/stirring

E: 25 g grape seed oil + 22.5 g water 15% + 1.455 Span 60 + 1.04 of Tween 80

F: 25 g grape seed oil + 22.5 g water 15% + 1.215 Span 60 + 1.285 of Tween 80

G: 25 g grape seed oil + 22.5 g water 15% + 0.975 Span 60 + 1.525 of Tween 80

H: 25 g grape seed oil + 22.5 g water 15% + 0.72 Span 60 + 1.77 of Tween 80

III) Tween 80/Span 60/homogenizer/5% emulsifier

I: 25 g grape seed oil + 22.5 g water 15% + 1.455 Span 60 + 1.04 of Tween 80

J: 25 g grape seed oil + 22.5 g water 15% + 1.215 Span 60 + 1.285 of Tween 80

K: 25 g grape seed oil + 22.5 g water 15% + 0.975 Span 60 + 1.525 of Tween 80

L: 25 g grape seed oil + 22.5 g water 15% + 0.72 Span 60 + 1.77 of Tween 80

IV) Tween 80/Span 60/homogenizer/2.5% emulsifier

M: 25 g grape seed oil + 23.75 g water 15% + 0.7275 Span 60 + 0.52 of Tween 80

N: 25 g grape seed oil + 23.75 g water 15% + 0.6075 Span 60 + 0.6425 of Tween 80

O: 25 g grape seed oil + 23.75 g water 15% + 0.4875 Span 60 + 0.7625 of Tween 80

### Assessment of stability, standardization, and phytochemical analysis of grape seed oils

Group III with the ratio of 40:60% with an emulsifier (5%) and group IV with the ratio of 40:60% with 2.5% emulsifier was evaluated for the monitoring of stability in a room (24 °C ± 1) and fridge (2 °C ± 1) temperatures.

The 1, 1-diphenyl-2-picrylhydrazyl radical (DPPH) is a stable radical with a maximum absorbance at 517 nm that can readily undergo reduction by an antioxidant. The scavenging effect on DPPH radical was determined by the method reported earlier with minor modifications [13]. Different concentrations of grape seed oil in methanol (20 μl) were mixed with 270 μl of 0.004% methanolic solution of DPPH. The mixture was shaken vigorously and left to stand for 30 min in dark at 30 °C, and the absorbance was then measured at 517 nm with a FLUostar OPTIMA spectrophotometer (BMG Labtech, Germany).

### Avocado extract preparation

Avocado fruits were obtained from a local market. Two hundred and fifty gram avocado slices were blended and macerated in 500 ml of alcohol (70% ethanol) at room temperature. The product was then filtered and the alcoholic extract was collected (the first portion). Subsequently, another 500 ml of ethanol was added to the residues, boiled for about 2 h under reflux condenser in a water bath, and then filtered. The filtrate was collected and added to the first portion. Together, these two portions formed the plant residue. Afterward, 500 ml of distilled water was added to the plant residue and left at room temperature for 2 days, and then filtered and added to the previous crude extract. Another volume of water was added to the residue, boiled for about 2 h under reflux condenser and filtered. Both products were gathered to form the hydro-alcoholic crude extract. The solvents were evaporated under vacuum by rotary evaporator. The extract was obtained and stored in a freezer until use.

### Induction of knee osteoarthritis and grouping

Seventy Sprague-Dawley male rats were used in this study, obtained from the animal center of Shiraz University of Medical Sciences. Animals were kept at 23 ± 2°C and a 12 h light:12 h dark cycle, 1 week before the experiment. On day 1 of the study, OA was induced based on a previously reported method through left anterior cruciate ligament transection under general anesthesia induced by intraperitoneal injection of a mixture of acepromazine and ketamine (1.25 mg/kg and 38 mg/kg, respectively) [14]. For preventing infection, tetracycline ointment was used after closing the surgical site.

Post-operatively, all 70 rats were randomly assigned into seven groups (*n* = 10): Group C1, negative control group with OA without any treatment; C2, positive control group receiving 300 mg/day of Piascledine dissolved in 1-2 cc distilled water (Expanscience Laboratories, France) per os (PO) daily from day 1 for 10 weeks; C3, second positive controls receiving 1 mg of sodium hyaluronate (Hyalgan®, Fidia Pharmaceutical, Padua, Italy) intra-articularly in the 1st, 7th, and 14th day; C4, positive control group receiving 1 mg methyl prednisolone acetate (Aburaihan Co, Tehran, Iran) intra-articularly in day 1, then in days 7 and 14; E1, the first experimental group receiving a combination of avocado and grape seed oil emulsion with a ratio of 2:1 (300 mg/day; dissolved in 1-2 cc distilled water) PO daily from day 1 for 10 weeks; E2, 500 mg/day of grape seed oil emulsion PO daily from day 1 for 10 weeks; E3 group treated with 200 mg/day grape seed oil injected intra-articularly in days 1, 7, and 14. All animals were controlled during 10 weeks of the study. The dosages of the medicines were chosen according to previously conducted pilot experiments and published papers showing the best efficacy with the lowest amount of the agent.

Ethical guidelines for animal studies were respected throughout the experiment period and the protocol was approved by the Ethics Committee of Shiraz University of Medical Sciences.

### Radiological and pathological examinations

Knee joint X-ray images were taken from anteroposterior and lateral aspects of the left knee. All radiographs were performed by the same operator and equipment and by using a standard protocol. OA was assessed by using a grading system based on the International Cartilage Repair Society (ICRS, Table 1) [15]. Scoring subjects were based on radiological features such as narrowing of the joint space, presence of osteophytes, sclerosis of subchondral bone, and deformity of the bone ends included 0 (none), 1 (doubtful), 2 (minimal), 3 (moderate), and 4 (severe). Osteophytes in the medial condyle of the tibia, femur, medial fabella, and total knee joint, joint space width, and total OA score, were investigated. Radiographs were graded by a blinded radiologist.

**Table 1:** Grading system of radiological assessment

Radiographic OA feature of the medial compartment		Grade 0	Grade 1	Grade 2	Grade3
Joint space width		Normal	Reduced	Absent	NA
Osteophyte	Medial tibial condyle	Absent	Small	Moderate	Severe
	Medial femoral condyle	Absent	Small	Moderate	Severe
	Medial fabella	Absent	Present		NA
Total osteophytes				0-7	
Global OA score				0-9	

For pathological evaluation, the joint surfaces were grossly examined after sacrificing the rats. Distal femora were removed and fixed in 10% buffered formaldehyde. All pieces were embedded in paraffin. Serial sagittal sections were prepared and stained with hematoxylin and eosin (for cellular architecture), toluidine blue, and safranin O (for proteoglycan contents of the matrix). A blinded pathologist performed the evaluation by using a modified histological grading method provided [16]. A scoring system was based on the following repair indices: surface, matrix, cell distribution, cell population viability, subchondral bone, and cartilage mineralization. More severe damage is indicated with a lower score. All morphometric parameters were recorded with a digital camera system (Olympus Optical, Tokyo, Japan).

### Statistical analysis

Gathered data were analyzed by the GraphPad software version 6.0 (San Diego, California). For histopathological and radiological evaluation, non-parametric Kruskal-Wallis and Mann-Whitney *U* tests were used through the SPSS software version 21.0 (IBM™, USA). A *P* value ≤ 0.05 was considered as statistically significant.

## Results

### Grape seed oils emulsification outcome

Regarding the composition of grape seed oil, in group I, samples A-D, each of the emulsions could not remain constant and two phases were produced. In the second experiment (group II, samples E-H), the Span 60 was used instead of Span 20. After 1 day, the emulsions turned into two phases. In the third experiment (group III, sample I-L), the homogenizer apparatus was used for mixing the lipid and aqueous layer. Interestingly, after 10 days, the emulsion did not turn into two phases. Conclusively, group III was chosen for further evaluations. Another critical factor that should be considered was sterilization. As a result, I-L was autoclaved at 121 °C for 15 min. The K and L samples became two phasic during sterilization and were removed. Finally, G and K with HLB:10 and ratio of 40:60% were chosen.

### Stability and phytochemical analyses outcomes

Studying the stability of the emulsion included droplet size determinations and viscosity measurements for a period of 1 day to 3 months in room and fridge temperatures. None of the emulsions of 5% with HLB = 10 exhibited creaming or phase separation during the abovementioned time period. Finally, samples containing 5% emulsifier with a ratio of 40:60% (lipid: aqueous) were selected in order to have more stability for biological evaluations.

The DPPH assay constitutes a quick and low-cost method that has frequently been used for evaluation of the anti-oxidative potential of various natural products. In the presence of antioxidant agents, the odd electron of DPPH becomes paired, resulting in the absorption loss. The absorbance change produced by this reaction is assessed to evaluate the antioxidant potential of the test sample. The results revealed that the IC50 scavenging effect of the avocado extract was 0.66 ± 0.1 mg/ml.

### Radiological findings

**Fig. 1:**
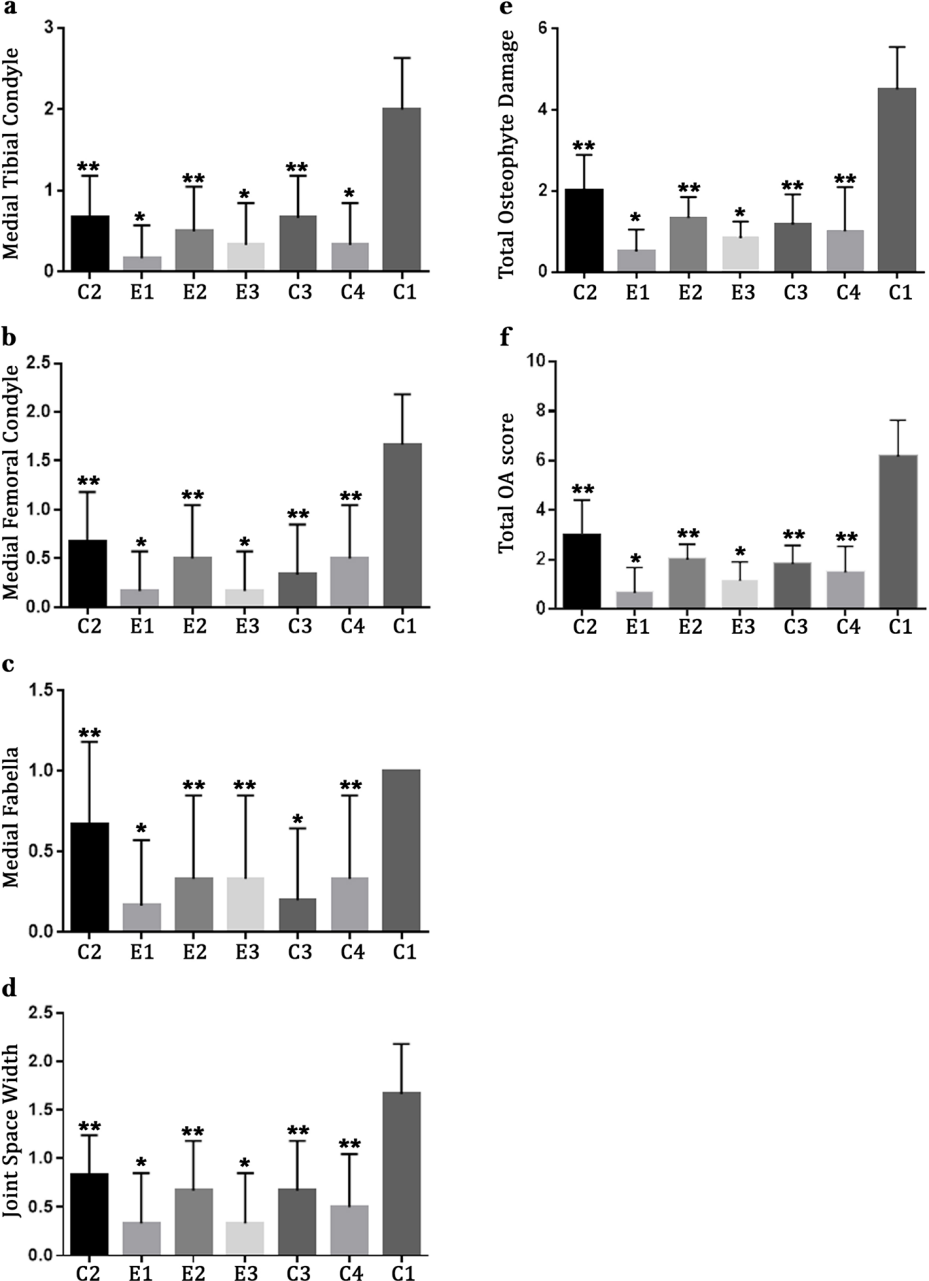
Radiological evaluations in rat models of OA based on ICRS scoring; data are indicated as mean ± standard deviation. Groups are named as C1, receiving no treatment; C2, receiving 300 mg/day of Piascledine per os (PO) for 10 weeks; C3, 1 mg sodium hyaluronate intra-articularly in days 1, 7, 14; C4, 1 mg methyl-prednisolone acetate intra-articularly in days 1, 7, 14; E1, avocado and grape seed oil combination (2:1, 300 mg/day) PO daily; E2, 500 mg/day of grape seed oil PO daily; E3, 200 mg/day grape seed oil intra-articularly in days 1, 7, 14. *P < 0.05 E1 and E3 versus other treatment groups. **P < 0.05 treatment group versus non-treated group (C1)

Our radiological findings, as shown in Fig. 1, depicted that all of the groups made significant differences with the untreated group C1 (*P* ≤ 0.05) regarding all inspected criteria in this study. Considering the findings of OA in the medial femoral condyle, joint space width, and total score for OA, groups E1 and E3 showed the lowest scores which significantly differed with the other groups (*P* ≤ 0.04). Scores of medial tibial condyle osteophytes were considerably lower in E1, E3, and C4 groups compared to the others (*P* ≤ 0.03), also, E1 and C3 groups depicted noticeably better results in medial fabella osteophytes (*P* ≤ 0.05). Regarding medial fabella osteophytes, total osteophyte, and total OA scores E1 group presented even better results than E3 (*P* ≤ 0.05; Fig. 1).

### Histopathological finding

**Fig. 2:**
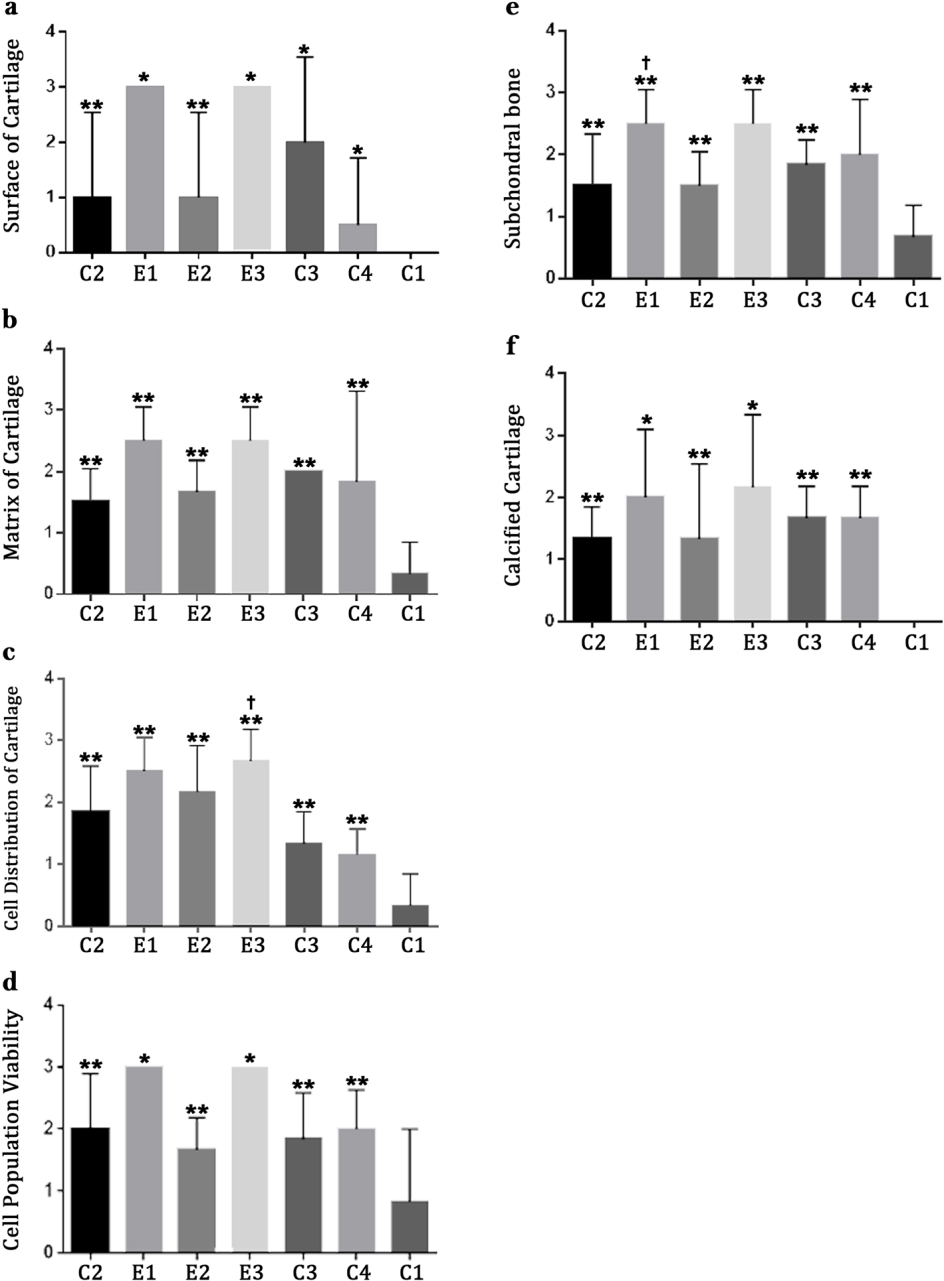
Pathological findings in rat models of OA; data are presented as mean ± standard deviation. Groups are named as C1, receiving no treatment; C2, receiving 300 mg/day of Piascledine per os (PO) for 10 weeks; C3, 1 mg sodium hyaluronate intra-articularly in days 1, 7, 14; C4, 1 mg methyl-prednisolone acetate intra-articularly in days 1, 7, 14; E1, avocado and grape seed oil combination (2:1, 300 mg/day) PO daily; E2, 500 mg/day of grape seed oil PO daily; E3, 200 mg/day grape seed oil intra-articularly in days 1, 7, 14. *P < 0.05 E1 and E3 versus other treatment groups. †*P* < 0.05 E1 or E3 versus other groups treated via the same route (PO or intra-articular). **P < 0.05 treatment group versus non-treated group (C1)

Pathological evaluation of joint surface, matrix, cell distribution, cell population viability, cartilage calcification, and subchondral bone showed that all treatment receiving groups had significantly better scores compared to C1 showing preventive effects of the treatments in rats with induced OA (*P* ≤ 0.04; Fig. 2). Groups E1 and E3 had significantly better results regarding joint surface, cell population viability, and cartilage calcification scores (*P* ≤ 0.04); E1 had considerably better results in regard to subchondral bone score compared to other PO treatments, and E3 showed noticeably lower score regarding cell distribution compared to other intra-articular treatments (*P* ≤ 0.05; Fig. 2).

**Fig. 3:**
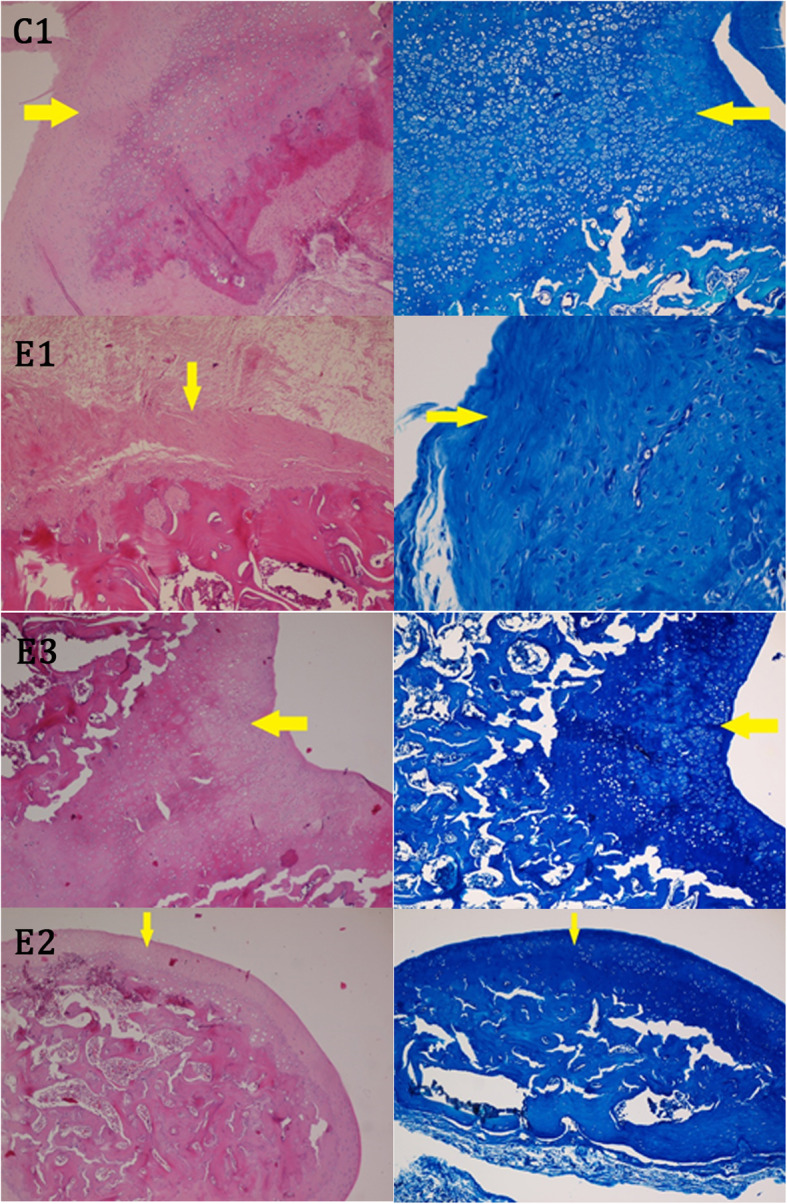
Samples of tissue sections from articular cartilage in groups C1, E1, E2, E3. Right, toluidine blue; left, hematoxylin and eosin (400×)

Evaluation of stained tissue sections of the C1 group presented irregular surface, fibrosis of the cartilage, and disrupted cell distribution with inflammation in the knee articular surface as well as mineralization of cartilage tissue (Fig. 3).

## Discussion

OA treatment includes medicinal therapy, non-medicinal therapy, and surgical procedures which are mostly focused on symptom therapies, pain reduction, and improving function overall [17, 18]. Historically, many natural remedies have been introduced as alternative treatments for OA. For instance, plants containing polyphenol components such as cocoa, grape, and pine bark, that through anti-inflammatory, immune modulating, and antioxidant activities can have positive effects on OA in terms of prevention or worsening of the symptoms [19, 20].

A number of studies that investigated the efficacy of grape seed oil on OA reported promising positive impacts. Woo et al. found that rats receiving grape seed oil had increased foot movement, reduced loss of chondrocytes, reduced inflammatory proteoglycans and cytokines, and reduced osteophyte formation, and concluded that grape seed oil can have protective effects on the devolvement of OA in knee joint in animal models [21]. Hailati et al. reported that procyanidins isolated from grape seed constrains the development of OA by their anti-oxidative impact, decreasing inflammatory cytokines such as IL-1B, and reducing osteophyte formation. Yet another experiment by Mevel et al*.* showed that oral administration of olive and grape seed extracts have anti-osteoarthritis effects due to their anti-oxidative and anti-inflammatory effects [19]. Similar results were found in an in vitro study by Jayaprakasha et al*.* who declared anti-oxidative and anti-inflammatory properties of grape seed oil play the main role regarding its anti-OA effects [22].

Our radiological and pathological studies suggest that intraarticular injection of grape seed oil, as well as oral administration of a combination of grape seed oil and avocado, have promising effects on OA. Its effects on preventing OA were more pronounced compared to routinely used Piascledine—which is a combination of avocado oil and soybean oil, in practice. Yet, intraarticular injection of grape seed oil alone showed better results in contrast with other common injectable medications. Overall, aiming to introduce this medication as an alternative treatment for OA, the efficacy of grape seed oil has to be evaluated in clinical studies and possible side effects must be investigated in short- and long-term surveys. However, since grape seed oil is widely used by local populations in many parts of Iran as well as some other countries in the region, proving its beneficial treatment for OA, as a cost-effective remedy in terms of a prevention or symptom reliever, can have a robust effect on the quality of life of these patients.

This study has a few limitations to consider. Though the antioxidative and anti-inflammatory effects of grape seed oil have been evaluated before we did not have the ability to evaluate parameters indicating these effects in our study and further biochemical studies are necessary in this regard. Despite that there are many similarities between knee OA in rats and its clinical features in humans, one should take note that these experimental models may not reproduce the full complexity of the human disease accurately. Not all factors associated with OA in humans are fully understood, and physiological biomechanics of the knee differ considerably between rodents and humans. Few in vivo studies have suggested the potential beneficial effects of grape seed oil on human health including cardioprotective, anti-inflammatory, antimicrobial, and anticancer properties [8, 12, 23]. However, possible side effects of grape seed oil, and the optimal dosage and application form to prevent disease have not been vastly explored. These unanswered questions also emphasize on the need for future in vivo research.

## Conclusion

Using a rat model, we found that grape seed oil, both in its intra-articular injectable form and in its oral administrative form had preventive effects for the development of knee OA. The efficacy of the combination of grape seed oil with avocado was comparable with Piascledine (avocado combination with soybean) which gives us the idea that the constituents of routinely used medications may change. Future in vivo research is required to investigate the potential preventive effects of grape seed oil in humans, as well as the optimal dosage, the application form, and possible side effects.

## Data Availability

The datasets used and/or analyzed during the current study are available from the corresponding author on reasonable request.

## Abbreviations

DPPH: 1-diphenyl-2-picrylhydrazyl radical
HLB: Hydrophilic-lipophilic balance
ICRS: International Cartilage Repair Society
IL: Interleukin
OA: Osteoarthritis
PO: Per os
